# Supplementary material for: Exploring the Surface: Sampling of Potential Skin Cancer Biomarkers Kynurenine and Tryptophan, Studied on 3D Melanocyte and Melanoma Models
Source: Biomolecules. 2024 Jul 9;14(7):815. doi: 10.3390/biom14070815 (PMC11274760; doi:10.3390/biom14070815)
Supplement: Supplementary file 1 [file biomolecules-14-00815-s001.zip › Supplementary materials_final_Proofread.pdf]

Article

Exploring the Surface: Sampling of Potential Skin Cancer Biomarkers Kynurenine and Tryptophan, Studied on 3D Melanocyte and Melanoma Models

Sylwia Hasterok [1,2,*], Skaidre Jankovskaja [1,2], Ruzica Miletic Dahlström [1,2], Zdenka Prgomet [1,2,3], Lars Ohlsson [1,2], Sebastian Björklund [1,2], and Anna Gustafsson [1,2,*]

[1] Department of Biomedical Science, Faculty of Health and Society, Malmö University, 205 06, Malmo, Sweden; skaidre.jankovskaja@mau.se (S. J.); ruzica.miletic@outlook.com (R.M.D); zdenka.prgomet@mau.se (Z. P.); lars.ohlsson@mau.se (L. O.); sebastian.bjorklund@mau.se (S. B.)

[2] Biofilms Research Center for Biointerfaces, Malmö University, 205 06, Malmo, Sweden

[3] Section for Oral Biology and Pathology, Faculty of Odontology, Malmö University, 214 21, Malmo, Sweden

* Correspondence: sylwia.hasterok@mau.se; anna.gustafsson@mau.se Tel.: +46-40-665-88-22 (S.H.); +46-40-665-74-16 (A.G.))

Supplementary Materials

Section 1 (S1). Simplified visualizations of mc and mm model reconstruction, stimulation, and sampling procedures.

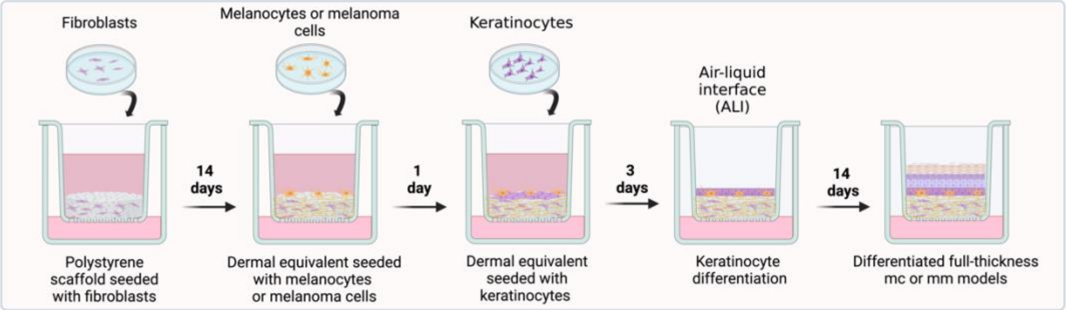

Figure S1. Simplified visualization of mc and mm model reconstruction procedure. The 3D models consisted of fibroblasts, melanocytes (mc) or melanoma cells (mm), and keratinocytes differentiated into distinct *strata* at air–liquid interface (ALI). The overall time of mc and mm model reconstruction was 32 days in total. Created with BioRender.com (accessed on 1 July 2024).

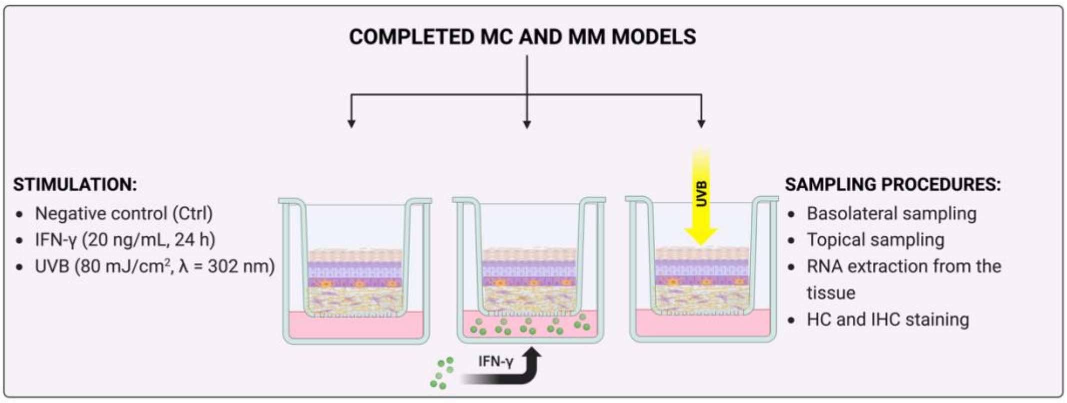

Figure S2. Simplified visualization of completed mc and mm model stimulation and types of sampling procedures. The models were subdivided into three treatment groups (Ctrl, IFN-γ, and UVB), followed by four sampling procedures (basolateral, topical, RNA extraction, and HC and IHC staining). HC—histochemical staining; IHC—immunohistochemical staining; λ—wavelength; Ctrl—Control. Created with BioRender.com (accessed on 1 July 2024).

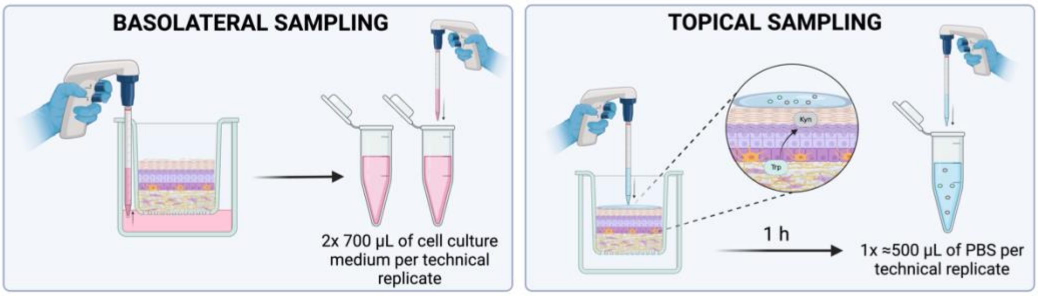

Figure S3. Simplified visualization of basolateral and topical sampling procedures. Basolateral sampling consisted of collecting 2× 700 µL of cell culture medium from each technical replicate of the mc or mm model and transferring the media into appropriate tubes. Topical sampling consisted of placing 500 µL of PBS onto the surface of each technical replicate of the 3D models, 1 h incubation, and transfer of ≈500 µL PBS into appropriate tubes. All samples were later stored at −80 °C until further use. Created with BioRender.com (accessed on 1 July 2024).

Section 2 (S2). Preparation of the standard solutions of Tyr, Phe, Trp and Kyn.

Stock solutions of Trp, 4.08 mg/mL (20 mM), Kyn, 4.16 mg/mL (20 mM), and Phe, 1 mg/mL (6.05 mM) were prepared in Milli-Q water. A stock solution of Tyr was prepared by dissolving in 99.9% formic acid and diluting it further with Milli-Q water to reach 1 mg/mL (5.52 mM) (final concentration of formic acid did not exceed 1% v/v). Fresh stock solutions of Trp, Kyn and Phe were kept at -20 °C for no longer than 2 weeks and used to prepare dilution series for HPLC-PDA or HPLC-MS calibration curves. Dilution series were prepared using the sample matrix, i.e., PBS or a relevant cell culture medium. Due to the instability and poor solubility of Tyr, a fresh stock solution was prepared each time HPLC-MS analysis was performed.

Table S1. Precision and accuracy of Trp and Kyn quantification by HPLC-PDA in cell culture medium. Calibration solutions were prepared in cell culture media used for models containing melanocytes (mc) or melanoma (mm) cells and measured in triplicates. The precision is defined as the CV (%) of these

measurements, i.e., CV = SD/Mean × 100%. Accuracy is reported as the recovery of the analytes where full recovery is defined as 100%.

Concentration (µM)	Precision (CV, %)				Accuracy (%)			
	mc		mm		mc		mm	
	Trp	Kyn	Trp	Kyn	Trp	Kyn	Trp	Kyn
0.78125	2.8	7.8	5.8	2.2	86	98	103	86
1.5625	8.8	2.3	5.1	1.6	93	103	97	96
6.25	1.2	0.7	5.5	1.2	103	102	103	101
25	0.7	0.4	3.7	0.7	101	100	100	101
50	0.5	0.3	1.6	0.4	99	100	99	100
100	0.4	0.5	1.7	0.6	100	100	100	100

Table S2. Selected ions monitored during HPLC-MS SIR experiments. Ions in bold were used for analytes quantifications (quantifiers).

Analyte	Selected ions (m/z)
Tyrosine (Tyr)	**182**, 166
Phenylalanine (Phe)	**166**, 120
Tryptophan (Trp)	205, **188**
Kynurenine (Kyn)	209, **192**

(a) Precision and accuracy of analytes quantification in PBS (topical samples).

Table S3. Precision and accuracy of Tyr, Phe, Trp, and Kyn quantification by HPLC-MS. Calibration solutions were prepared in PBS for Tyr, Phe, Trp, and Kyn, and measured for three consecutive days. The precision is defined as the CV (%) of these measurements, i.e., CV = SD/Mean × 100%. Accuracy is reported as the recovery of the analytes where full recovery is defined as 100%.

Concentration (µM)		Precision (CV, %)				Accuracy (%)			
**Phe, Trp, Kyn**	**Tyr**	Tyr	Phe	Trp	Kyn	Tyr	Phe	Trp	Kyn
0.15625	0.46875	6	10	5	8	149	98	70	108
0.3125	0.9375	9	11	4	7	97	98	95	100
0.625	1.875	10	11	4	7	97	100	99	97
1.25	3.75	22	11	5	8	95	101	103	99
2.5	7.5	11	10	4	7	100	100	103	101
5	15	10	10	5	7	100	100	99	100

Section 3 (S3). Scatter plots illustrating the relationship between Kyn and Trp concentrations in the topical and basolateral samples of 3D skin models.

(a) Topical samples

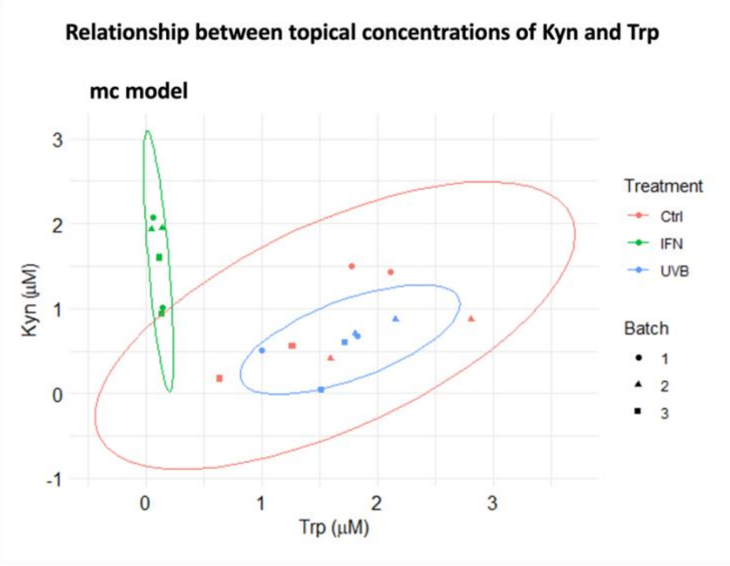

Figure S4. Scatter plot demonstrating the relationship between tryptophan (Trp) concentration (x-axis) and kynurenine (Kyn) concentration (y-axis) in topical samples of the mc model. Data are categorized by three treatment groups (Ctrl, IFN-γ, and UVB) and across three distinct batches (1-3), highlighting potential variations and trends within the model. T-distribution ellipses are overlaid to highlight clustering within each treatment group.

(b) Basolateral samples

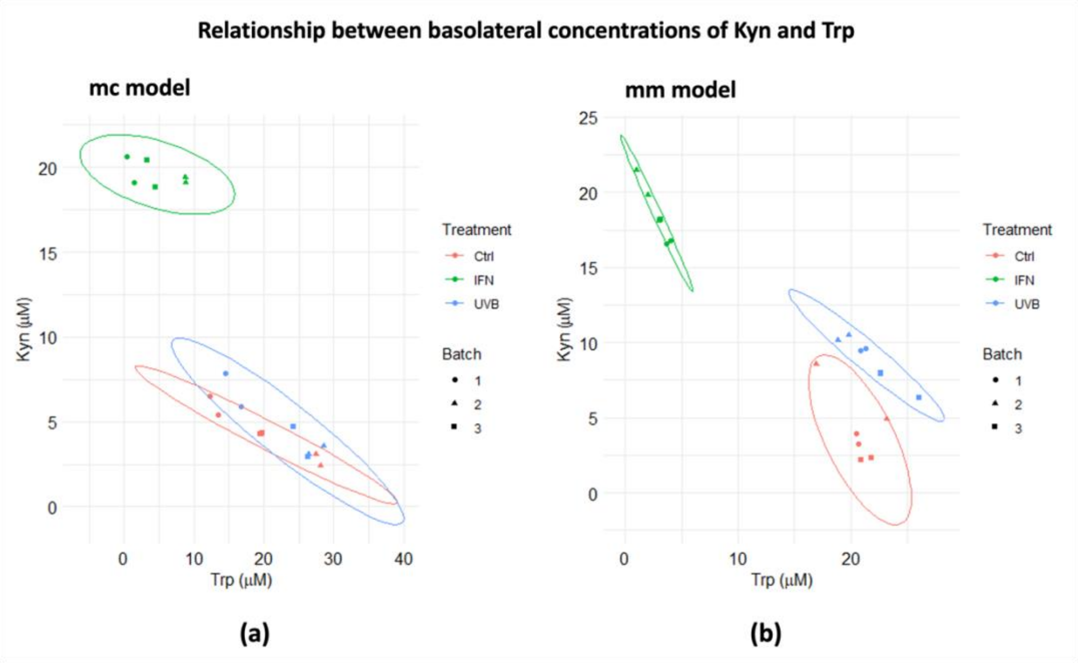

Figure S5. Scatter plots demonstrating the relationship between tryptophan (Trp) concentration (x-axis) and kynurenine (Kyn) concentration (y-axis) in basolateral samples of the (a) mc model and (b) mm model. Data are categorized by three treatment groups (Ctrl, IFN-γ, and UVB) and across three distinct batches (1–3), highlighting potential variations and trends within the models. T-distribution ellipses are overlaid to highlight clustering within each treatment group.
